# Effect of Mineral-Balanced Deep-Sea Water on Kidney Function and Renal Oxidative Stress Markers in Rats Fed a High-Salt Diet

**DOI:** 10.3390/ijms222413415

**Published:** 2021-12-14

**Authors:** So Min Jo, Jain Nam, Soo-yeon Park, Geonhee Park, Byeong Goo Kim, Gwi-Hwa Jeong, Byung Serk Hurh, Ji Yeon Kim

**Affiliations:** 1 Department of Food Science and Technology, Seoul National University of Science and Technology, Seoul 01811, Korea; mindar71@naver.com (S.M.J.); jain3513@naver.com (J.N.); 2 Department of Nano Bio Engineering, Seoul National University of Science and Technology, Seoul 01811, Korea; sooyeon.park@seoultech.ac.kr; 3 Sempio Fermentation Research Center, 183, Osongsaengmyeong 4-ro, Osong-eup, Heungdeok-gu, Cheongju-si 28156, Chungcheongbuk-do, Korea; pgeonhee@sempio.com (G.P.); kbyeonggoo@sempio.com (B.G.K.); jgwihwa@sempio.com (G.-H.J.); hbyungserk@sempio.com (B.S.H.)

**Keywords:** deep sea water, kidney, high-salt diet, antioxidant, kidney injury, sodium excretion, magnesium, calcium, RNA-seq

## Abstract

This study investigated the effect of mineral-balanced deep-sea water (DSW) on kidney health using an animal model of kidney injury due to a high-sodium diet. High magnesium/low sodium (HMLS) and high magnesium/high calcium (HMHC) DSW samples with different mineral contents were prepared. Sprague–Dawley rats were fed an 8% sodium chloride (NaCl) diet for four weeks to induce kidney injury, and each group was supplied with purified water or mineral water. Kidney injury was observed in the NaCl group according to increased kidney injury markers and malondialdehydes, providing evidence of oxidative stress. However, the kidney injury was repaired by the intake of mineral-balanced DSW. It was confirmed that the HMLS and HMHC groups showed improved Na^+^ excretion through the urine. Kidney injury markers in urine decreased and upregulation of low-density lipoprotein receptor-related protein2 mRNA expression was observed in the HMLS and HMHC groups. In addition, superoxide dismutase activity was increased in the HMHC groups. The gene expression patterns of the RNA sequencing were similar between the CON and HMLS groups. These results suggest that DSW has beneficial effects on kidney health due to the balanced magnesium and calcium levels in models of kidney injury caused by excessive sodium intake.

## 1. Introduction

At present, people are consuming more salt in their diets. The general sodium intake of adults in the United States continues to exceed the chronic disease risk reduction (CDRR) intake guidelines established by considering the association between sodium intake and the risk of high blood pressure [1]. The adverse effects of a high-salt diet have been demonstrated in many studies. A high salt intake increases blood pressure, leading to hypertension and possibly even heart failure [2]. In addition, it has been reported that high salt intake levels increase the possibility of metabolic syndromes such as obesity and type 2 diabetes [3].

The kidneys play a crucial role in maintaining our health. They regulate the homeostasis of minerals such as calcium (Ca), magnesium (Mg), phosphorus (P), and sodium (Na) through filtration and reabsorption processes [4,5]. Fellner et al. [6] have shown that high-salt diet blunts renal blood flow (RBF) and glomerular filtration rate (GFR). There have been many studies of the relationship between a high salt intake and kidney injury, confirming that a high-salt diet causes renal tubular injury, renal fibrosis, and deteriorates the pathophysiological renal function [7,8]. Kidney injuries, when not treated, can progress to kidney failure and can have catastrophic effects such as cardiovascular disease [9]. In sum, kidney injuries associated with a high salt diet can affect kidney function, such as filtration and reabsorption processes. In addition, impaired kidney function is associated with reactive oxygen species (ROS). In rats, a high-salt diet increases ROS production and induces dysfunction of mitochondria in the kidneys [10], and decreased GFR via NADPH oxidase-derived ROS generation [11].

Minerals are essential for biological processes in organisms and help to keep the body functioning properly. They play a vital role in animal metabolism by participating as an enzyme cofactor, contributing to proper cellular and tissue function, and even improving the immune system [12,13]. It has been discovered that the supplementation of magnesium can help to decrease oxidative stress and recover antioxidant levels by synthesizing glutathione and vitamin C [14]. Deep sea water (DSW) is known to have numerous beneficial effects on health, as it contains various minerals, such as Ca, Mg, selenium (Se), and zinc (Zn) [15]. Accordingly, there have been many attempts to process this water as drinking water. Research on the beneficial effects on health of DSW is ongoing.

Previously, we confirmed the effects of mineral-balanced DSW on human embryonic kidney (HEK293) cells exposed to sodium chloride (NaCl) [16]. This line of research was continued in a study involving an animal model fed a high-salt diet using two DSW samples found to be most effective in reducing oxidative stress in HEK293 cells. Meanwhile, many studies have investigated kidney damage due to high salt levels, but there are few studies, to the best of our knowledge, about how mineral-balanced DSW affects the kidneys in a high-salt model. Experiments were performed to determine whether DSW could protect kidney function by promoting sodium excretion or ameliorating oxidative stress. In addition, we checked whether both mineral-balanced DSW with different magnesium and calcium contents can help protect kidney function.

Sprague–Dawley rats were fed an 8% NaCl diet for four weeks to induce kidney injury, and each group was given purified water or mineral-balanced water as drinking water. After four weeks, clinical parameters and biomarkers were measured to confirm the effects of DSW on kidney injury. This study also provided an analysis of RNA levels with regard to the effects of DSW on high-salt-induced kidney injury.

## 2. Results

### 2.1. Clinical Symptoms

Data pertaining to the clinical symptoms are shown in Table 1. No weight loss or increase of more than 5% was observed in the NaCl group and the sample groups compared to the CON group. A decrease in food intake was observed at the first day of experiment due to the replacement of 8% NaCl feed in the NaCl, HMLS, and HMHC groups. Then, the food intake gradually increased, and no significant differences in food intake between all groups were observed in the 4th week. On day 28 of the test period, a 131% increase in the water intake of the NaCl group was noted compared to the CON group, while the water intake levels of the HMLS and HMHC groups increased by 31.3% and 23.0%, respectively, compared to the NaCl group. Kidney weight was increased in the NaCl, HMLS and HMHC groups compared to the CON group. There was no significant difference between the NaCl, HMLS and HMHC groups.

The systolic blood pressure of the CON group was 106.9 ± 8.4 mmHg ~ 113.1 ± 4.6 mmHg on average during the experimental period and remained below the normal value of 120 mmHg. In the NaCl group, it increased by 30.1% compared to the CON group and the HMLS and HMHC groups showed no significant differences compared to the NaCl group.

### 2.2. Urine Volume, Sodium Excretion, and Urine Protein

**Figure 1:**
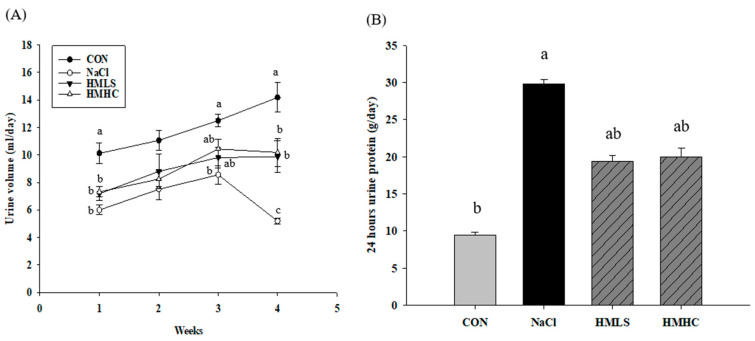

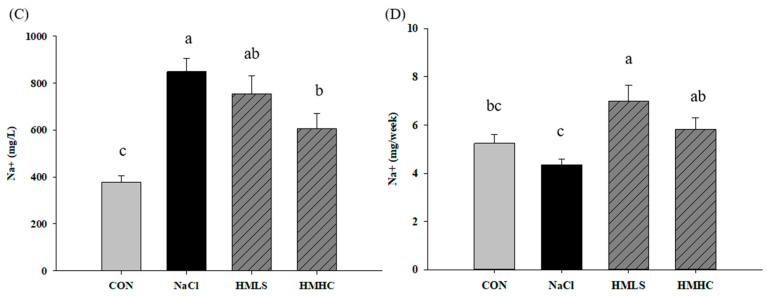
Effect of mineral-balanced DSW on the kidneys through a urine analysis: (**A**) urine volume at four weeks, (**B**) urine protein during the fourth week, (**C**) sodium excretion in the urine during the fourth week, and (**D**) sodium excretion in the urine in the fourth week corrected according to the urine volume. CON, normal diet + distilled water; NaCl, 8% NaCl diet + distilled water; HMLS, 8% NaCl diet + high magnesium low sodium water; HMHC, 8% NaCl diet + high magnesium high calcium water. Bonferroni test was applied to urine volume. Other data were evaluated using Duncan’s multiple range test. Different letters above the bars indicate significant differences (*p* < 0.05).

Figure 1 shows the influence of mineral water on the kidneys through the urine volume, urine protein levels, and sodium excretion in the urine. The urine volume increased until the third week in all groups but dropped at the fourth week for the NaCl group. During the fourth week, the urine volume of the NaCl group decreased by 63.44% compared to that of the CON group. In the HMLS and HMHC groups, the urine volume increased by 90.36% and 96.39%, respectively, compared to the NaCl group (Figure 1A). It was confirmed that the intake of HMLS and HMHC decreased the reduction of the urine volume initially created by a high-salt diet.

The amount of urinary protein was increased by 216.88% in the NaCl group compared to that in the CON group. In the mineral water groups, urinary protein excretion was reduced by 34.94% in the HMLS group and 33.06% in the HMHC group compared to the NaCl group (Figure 1B). This result suggests that mineral water reduced urine protein excretion stemming from a high-salt diet.

The concentration of sodium in the urine is shown in Figure 1C. Figure 1D presents values obtained by correcting the amount of sodium excretion according to the urine volume. Sodium excretion in the NaCl group was decreased 17.18% compared to the CON group. However, compared to the NaCl group, sodium excretion increased significantly by 61.08% in the HMLS group and 34.03% in the HMHC group. It was shown that mineral water increased sodium excretion through urine.

### 2.3. Kidney Injury Markers

**Figure 2:**
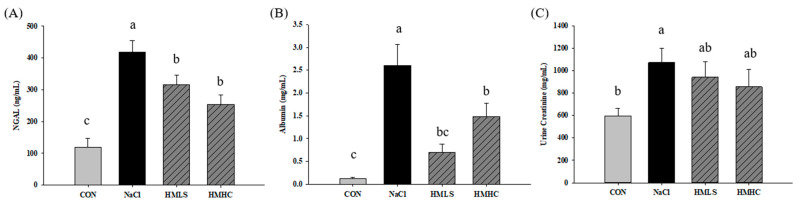
Effects of mineral-balanced DSW on kidney injury markers measured in urine: (**A**) NGAL, (**B**) albumin, and (**C**) creatinine. CON, normal diet + distilled water; NaCl, 8% NaCl diet + distilled water; HMLS, 8% NaCl diet + high magnesium low sodium water; HMHC, 8% NaCl diet + high magnesium high calcium water. Different letters above the bars indicate significant differences (Duncan’s multiple range test; *p* < 0.05).

The effect of mineral water on kidney function was confirmed by analyzing acute kidney injury markers, in this case, neutrophil gelatinase-associated lipocalin (NGAL), albumin, and creatinine in the urine. In the NaCl group, NGAL increased by 252.43% over the control group. The level of NGAL showed significant decreases, 24.28% in the HMLS group and 39.08% in the HMHC group compared to the NaCl group (Figure 2A). NaCl group showed increased levels of urinary albumin and creatinine compared to the CON group. Albumin in the HMLS group declined by 72.94%, and it declined by 43.09% in the HMHC group, a significant level compared to the NaCl group (Figure 2B). Mineral water slightly reduced urine creatinine levels by 12.22% in the HMLS and 20.14% in the HMHC groups (Figure 2C).

### 2.4. Relative mRNA Expression of Genes Related to Kidney Function

**Figure 3:**
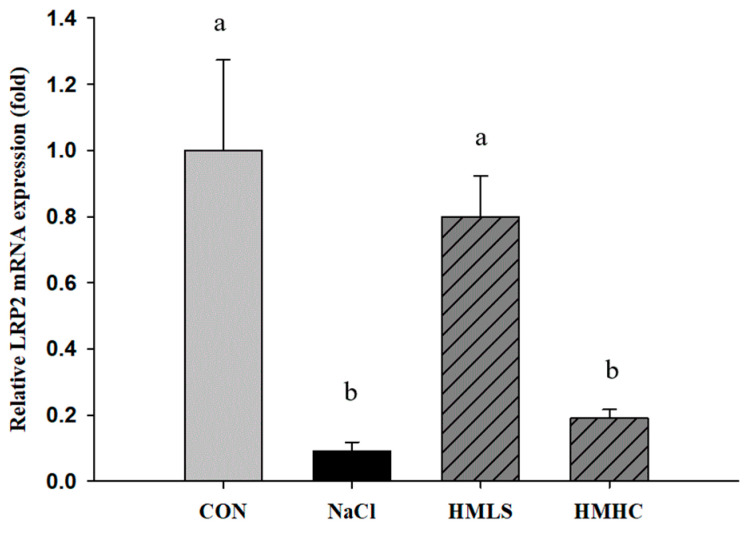
Effects of mineral-balanced DSW on low-density lipoprotein-related protein 2 (LRP2) expression levels: CON, normal diet + distilled water; NaCl, 8% NaCl diet + distilled water; HMLS, 8% NaCl diet + high magnesium low sodium water; HMHC, 8% NaCl diet + high magnesium high calcium water. Different letters above the bars indicate significant differences (Duncan’s multiple range test; *p* < 0.05).

LRP2 mRNA expression was significantly decreased due to NaCl intake. However, the consumption of HMLS significantly increased the LRP2 mRNA expression level, and there were no significant differences in the HMHC group (Figure 3).

### 2.5. Oxidative Stress and Antioxidant Activity

**Figure 4:**
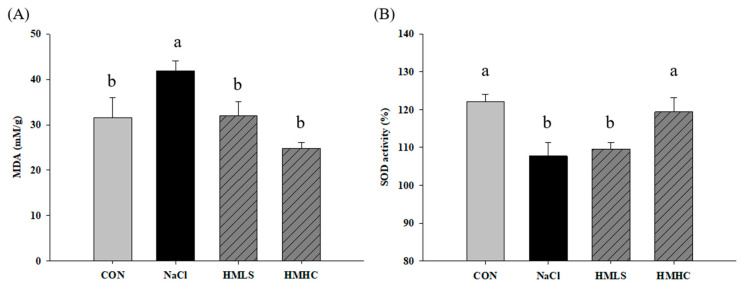
Effects of mineral-balanced DSW on antioxidant effect in kidney tissue: (**A**) malondialdehyde, and (**B**) SOD activity (%). CON, normal diet + distilled water; NaCl, 8% NaCl diet + distilled water; HMLS, 8% NaCl diet + high magnesium low sodium water; HMHC, 8% NaCl diet + high magnesium high calcium water. Different letters above the bars indicate significant differences (Duncan’s multiple range test; *p* < 0.05).

Figure 4 shows the effect of mineral water on oxidative stress and antioxidant activity in kidney tissue. Oxidative stress can be confirmed by MDA level. The MDA value was increased by 32.65% in the NaCl group compared to that in the CON group. However, it was also found that mineral water significantly reduced MDA levels. Compared to the NaCl group, the level of MDA was decreased by 23.80% in the HMLS group and by 41.00% in the HMHC group (Figure 4A).

SOD activity was 122.02% in the CON group and was decreased by 11.70% in the NaCl group compared to the CON group. For the HMHC group, it increased significantly by 10.81% compared to the NaCl group. However, there was no significant difference in the HMLS group (Figure 4B).

### 2.6. Relative mRNA Expression of Antioxidant Genes

**Figure 5:**
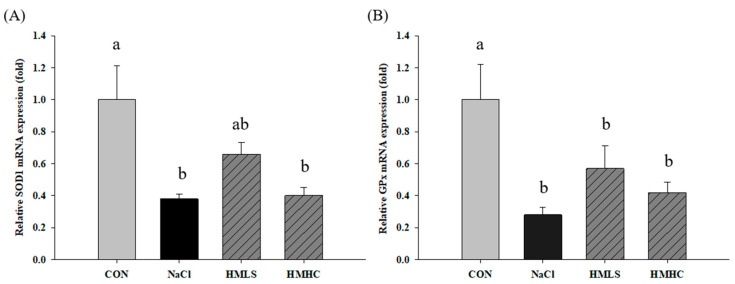

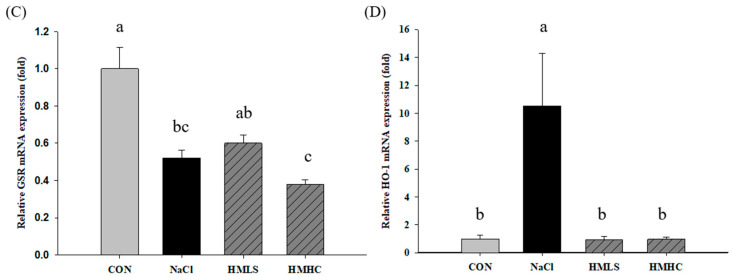
Effects of mineral-balanced DSW on antioxidant related mRNA expression levels of (**A**) superoxide dismutase 1 (SOD1), (**B**) glutathione peroxidase (GPx), (**C**) glutathione disulfide reductase (GSR), and (**D**) heme oxygenase-1 (HO-1). CON, normal diet + distilled water; NaCl, 8% NaCl diet + distilled water; HMLS, 8% NaCl diet + high magnesium low sodium water; HMHC, 8% NaCl diet + high magnesium high calcium water. Different letters above the bars indicate significant differences (Duncan’s multiple range test; *p* < 0.05).

To confirm the exact pathway of the antioxidant effect of mineral-balanced DSW water, the mRNA expression levels of the antioxidant-related genes were examined (Figure 5). Compared to the CON group, superoxide dismutase 1 (SOD1), glutathione peroxidase (GPx), and glutathione disulfide reductase (GSR) mRNA expression levels were significantly decreased in the NaCl group. However, SOD1 and GSR mRNA expression levels showed increasing tendency in the HMLS group compared to the NaCl group, whereas there were no differences in the HMHC group. Regarding the GPx mRNA expression outcomes, they showed slight increases, albeit not at significant levels, in both the HMLS and HMHC groups. On the other hand, heme oxygenase-1 (HO-1) mRNA expression level was significantly increased in the NaCl group compared to that in the CON group. The corresponding values were significantly decreased in the HMLS and HMHC groups. This tendency was in agreement with the differentially expressed genes (DEG) result from RNA sequencing.

### 2.7. Total RNA-Sequencing and Differentially Expressed Gene Analysis

**Figure 6:**
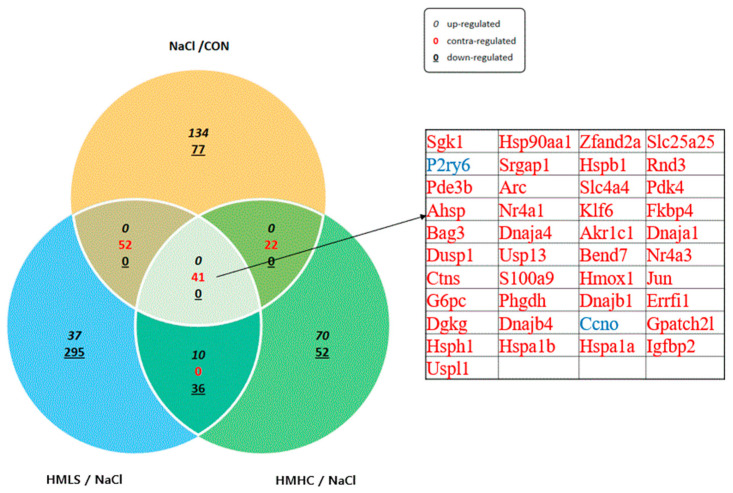
Examination of differentially expressed genes in kidney tissues. CON, normal diet + distilled water; NaCl, 8% NaCl diet + distilled water; HMLS, 8% NaCl diet + high magnesium low sodium water; HMHC, 8% NaCl diet + high magnesium high calcium water. Differentially expressed genes screened from the kidneys of rats. Genes that satisfied the criteria of fold change ≥ 2, normalized data (log2) = 0, and *p*-value ≤ 0.05 were selected for CON vs. NaCl, HMLS vs. NaCl, and HMHC vs. NaCl.

Differentially expressed genes (DEGs) were analyzed using the ExDEGA tool provided by EBiogen. In this case, 17,048 genes were screened from the kidneys of rats, of which the fold change ≥ 2, normalized data (log2) = 0, and *p*-values ≤ 0.05 were selected for CON vs. NaCl, HMLS vs. NaCl, and HMHC vs. NaCl. The total number of DEGs in CON vs. NaCl was 326, of which 233 were up-regulated and 93 were down-regulated. The total number of DEGs in HMLS vs. NaCl was 471, of which 56 were up-regulated and 415 were down-regulated. The total number of DEGs in HMHC vs. NaCl was 231, of which 89 were up-regulated and 142 were down-regulated. In the three comparison groups, 41 genes had a common difference in their expression values. The 41 genes whose expression values were significantly different in all three comparison groups are listed in Figure 6.

**Figure 7:**
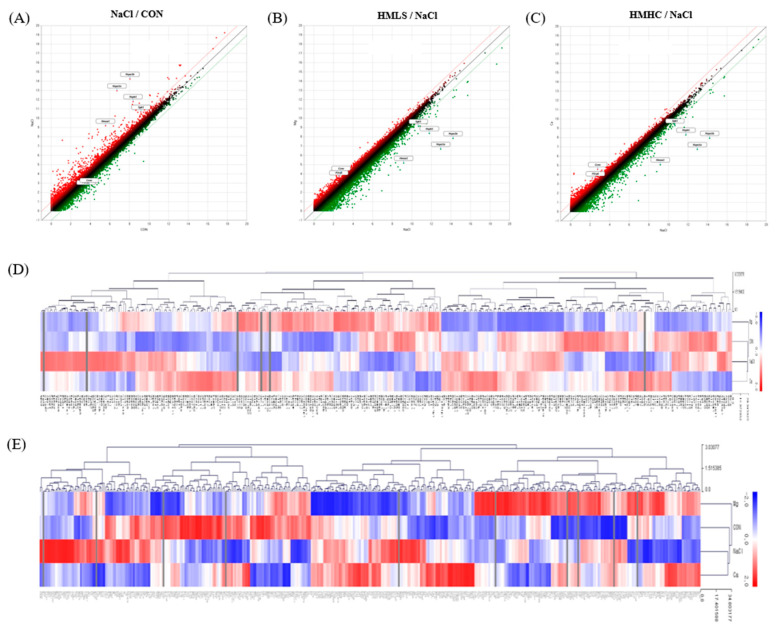
Differentially expressed genes in kidney tissues: (**A**–**C**) Scatter plots showing the correlation between the mRNA expression level in CON vs. NaCl, HMLS vs. NaCl, and HMHC vs. NaCl. (**D**) Heatmap hierarchical clustering with 344 differentially expressed genes related to renal function. (**E**) Heatmap hierarchical clustering with 424 differentially expressed genes related to the response to oxidative stress. CON, normal diet + distilled water; NaCl, 8% NaCl diet + distilled water; HMLS, 8% NaCl diet + high magnesium low sodium water; HMHC, 8% NaCl diet + high magnesium high calcium water.

The expression patterns between the groups were confirmed as an image through scatter plots (Figure 7A–C). The genes above the red diagonal line are those whose fold change increased by two-fold or more, and the genes below the green line are those for which the fold change decreased by two-fold or more.

From the 17,048 genes in total, 424 genes were selected corresponding to the “response to oxidative stress (GO:0006979),” and 344 genes corresponding to the category related to renal function, such as “renal system development (GO:0072001)” and “renal sodium excretion (GO:0035812)” were also selected by means of Gene Ontology and Go annotation (https://www.ebi.ac.uk/QuickGO (accessed on 6 December 2021)).

A hierarchical clustering heatmap was drawn using the MeV program to determine the degree of expression similarity between the samples (Figure 7D,E). The length of the hierarchical branch indicates the degree of expression similarity between the groups: the shorter this distance is, the more similar the levels are. Regarding the heatmap related to the renal function category, the expression patterns were similar between the NaCl and HMHC groups, and the HMLS group showed the expression pattern most similar to that of the CON group. Here, 15 genes were expressed differently in NaCl vs. HMLS, with hemoglobin subunit beta (Hbb) and nephrosis 2 idiopathic steroid-resistant (Nphs2) also down-regulated to 0.238 and 0.240-fold, respectively (Table 2). In the heatmap of the “response to oxidative stress” category, the NaCl and HMHC groups showed similar expression patterns, with the CON group showing the expression pattern most similar to that of the HMLS group. There were 13 genes whose expression levels were significantly changed in NaCl vs. HMLS. Above them, heme oxygenase-1 (HO-1) was the most down-regulated gene, with a fold change of 0.062 (Table 3).

### 2.8. GO and KEGG Enrichment Analyses of DEG

In the NaCl vs. HMLS results, 471 genes whose expression values were changed were analyzed by means of a DAVID analysis to determine their function and pathway. In the GO cellular component, they were grouped as follows: “Hemoglobin complex”, “Perinuclear region of cytoplasm” and “Extracellular exosome”. They were also grouped into categories entitled “Negative regulation of inclusion body assembly”, “Oxygen transport” and “Negative regulation of neuron apoptotic process” with respect to the GO biological process. An additional grouping was “Unfolded protein binding”, “Chaperone binding” and “Oxygen transporter activity”. In the KEGG pathway, they were grouped according to the “MAPK signaling pathway”, “African trypanosomiasis” and the “Estrogen signaling pathway”. These results demonstrated that there were many genes related to oxygen transport (Table 4).

## 3. Discussion

Modern people who consume a high-salt diet are prone to various diseases, and the kidneys, which are important organs that play a role in the removal of waste from our bodies and in regulating water, salt, electrolytes, and maintaining the acid-base balance in our bodies, can also be affected by a high-salt diet, possibly leading to kidney injury. We confirmed that mineral-balanced DSW can effectively reduce kidney injury caused by alleviating oxidative stress and that it can help with the excretion of sodium in the kidneys through a model in which rats were fed a high-salt diet to induce kidney injury.

The renin-angiotensin-aldosterone system (RAAS) contributes to kidney injury [17] and is stimulated when the body receives excessive salt [18]. Angiotensinogen is cut by renin and forms angiotensin Ι (Ang Ι), after which it turns into angiotensin ΙΙ (Ang ΙΙ) by the actions of angiotensin-converting enzyme (ACE). Ang ΙΙ is responsible for producing aldosterone, and aldosterone is known to induce injury to kidney tissues and is involved in podocyte injury, fibrotic processes, and renal inflammation [17,19]. ROS are produced in the kidney in response to aldosterone [19]. In the case of inappropriate sodium intake or balance, aldosterone can activate NADPH oxidase which is a source of ROS [20]. Therefore, a high-salt diet induces kidney injury by increasing oxidative stress [21].

Oxidative stress can cause proteinuria, which is an important factor in defining chronic kidney disease [22]. Conventional markers of kidney injury such as albumin and creatinine are widely used but sometimes show lower sensitivity levels. There are several novel markers of kidney injury, including vanin-1, kim-1 and NGAL, and it is known that NGAL increases more rapidly than urinary albumin [7,23]. In this study, we examined urinary albumin, creatinine, and NGAL as kidney injury markers and verified the effects of mineral water on kidney injury in terms of both conventional markers and novel markers.

Megalin, or low-density lipoprotein-related protein 2 (LRP2) is a transmembrane protein that functions as a receptor of multiple ligands [24]. In the kidney, megalin plays a central role by reabsorbing filtered molecules, including albumin and NGAL, at the proximal tubules [23,25]. When the kidney is damaged, megalin is harmed, and the mRNA expression of megalin can be down-regulated, resulting in proteinuria [26,27]. Washino [23] also reported that an 8% NaCl diet can reduce the mRNA expression of megalin in WKY rats, suggesting that this megalin damage reduces the reabsorption of NGAL. In our study, the mRNA expression of LRP2 was decreased by NaCl intake but was rescued by the consumption of HMLS. This result implies that mineral-balanced DSW repaired harmed transmembrane protein receptors. In addition, this repair resulted in decreased levels of urinary proteins.

This study measured Na^+^ excretion in the urine during the fourth week and corrected it according to the urine volume. We confirmed that Na^+^ excretion was reduced by NaCl, whereas DSW increased it. Other studies reported that supplementation of calcium and magnesium will increase sodium excretion through the urine [28,29]. From these results, it can be inferred that DSW can help to prevent kidney injury by reducing sodium remaining in the body by helping it be excreted through the urine.

It is known that continuous natriuresis to maintain sodium balance is accompanied by an increase in blood pressure [30]. The renal excretion capacity and blood pressure follow the following compensatory system. When arterial pressure rises above the normal range, the increased pressure increases the excretion of water and salt from the kidneys. As a result, blood volume decreases, followed by a decrease in blood pressure [30,31,32]. Rats in the NaCl, HMLS and HMHC groups drank more water due to NaCl intake, and their blood pressure increased. In the HMLS and HMHC groups, sodium excretion through urine was achieved, but blood pressure was still increased. Therefore, the compensation system was not applied well. There are mechanisms to control blood pressure, such as the renin-angiotensin system, however, as shown in the discussion, there was no significant change observed in aldosterone level. HMLS and HMHC may help with immediate sodium excretion but appears to be insufficient to alter blood pressure control mechanisms.

A previous study showed that magnesium supplementation lowered oxidative stress in diabetic rats in relation to decreased MDA levels, revealed that Mg is associated with the restoration of antioxidant levels and decreased oxidative stress by way of directly removing free radicals [14]. On the other hand, NADPH oxidase acts as a major contributor of ROS generation [33,34]. Although NADPH oxidase-related experiments were not conducted in our study, it is expected that magnesium contained in mineral-balanced water may play a role in inhibiting NADPH oxidase according to the research results showing that magnesium inhibits NADPH oxidase enzymes [35]. Gao [36] reported that mineral-rich salt generates less oxidative stress than mineral-deficient salt in rats. According to our findings, MDA was decreased by HMLS and HMHC and SOD activity was increased by HMHC, suggesting that mineral-balanced DSW acts as an effective antioxidant. It is assumed that Mg in DSW reduces oxidative stress by repressing free radical production [37], scavenging free radicals directly [38], and increasing SOD activity [38,39]. Furthermore, it has been reported that a high-calcium diet increases SOD activity in the kidneys and protects the kidneys from injury by reducing oxidative stress [40]. We also sought to confirm that supplements of DSW regulate the mRNA expression of antioxidant-related genes. SOD1 converts superoxide anion into hydrogen peroxide and oxygen, and the hydrogen peroxide produced here is decomposed into water and oxygen through CAT [41]. GPx (glutathione peroxidase) converts reduced glutathione (GSH) to oxidized glutathione (GSSG) while removing hydrogen peroxide via NADP^+^, and GSR (glutathione reductase) acts in the opposite way to regenerate GSH, an antioxidant protein. It is an enzyme that aids in antioxidant activity [42,43]. However, we did not clarify why the SOD activity is not increased by HMLS.

Following RNA-seq, among the 13 genes involved in the “response to oxidative stress” between the NaCl and HMLS groups, Hmox1 was the most down-regulated gene in the HMLS group compared to the NaCl group. HO-1 is induced in response to kidney injury and regulates oxidative stress [44]. We investigated DEGs with the opposite trend of an increase or decrease in NaCl compared to CON, and in HMLS or HMHC compared to the NaCl. In line with our findings, Zhang [45] reported that heat shock protein, heme oxygenase-1, and S100 calcium-binding proteins genes were upregulated during an ischemia-reperfusion injury in rat kidneys. Specifically, heat shock protein upregulation is assumed to be a physiological reaction to protect the kidney from cellular damage [45]. Nphs2 encodes a podocyte protein, podocin [46,47]. We assumed that the reason for the upregulation of Nphs2 in the NaCl group is to provide a protective response against podocyte injury due to high levels of salt.

In the DAVID analysis, we found that many genes were related to oxygen-carrying pathways, including the Hbb gene. Hbb mRNA expression is up-regulated by oxidative stress, and this can serve as an inhibitor of oxidative damage [48]. Our findings clearly demonstrate that oxidative stress was induced by a high-salt diet in the NaCl group and demonstrate an antioxidant effect of mineral-balanced DSW in the HMLS and HMHC groups. It is assumed that mineral-balanced DSW prevents the kidneys from being damaged by regulatory genes related to oxygen transport.

The positive effect of mineral water may be influenced not only by Mg and Ca but also by Mg/Ca or Na/Ca ratios. Many studies have investigated ideal ratios of minerals and the purported positive effect on health [49,50]. According to our study, we propose a ratio of 4.3 or 1.1 for Mg/Ca as effective for health. However, further study is needed to discover a clear mechanism by which this proposed Mg/Ca ratio work.

In this study, we examined the effects of mineral-balanced DSW on kidney health through high-salt diets fed to rats. This study provides various types of proof of the protective effect of mineral-balanced DSW on the kidneys. Specifically, we found that mineral-balanced DSW restores kidney injury through the excretion of Na^+^ via the urine and a reduction of oxidative stress. This study also suggests an ideal ratio of mineral-balanced DSW. However, this study has several limitations. We examined GPx, GSR and LRP2 mRNA expressions but did not verify them at the protein level. The lack of western blotting or immunohistology data and inflammatory markers are limitations of this study. Although DSW was expected to reduce aldosterone and blood pressure levels by regulating how RAAS affects renal injury, no significant changes were detected (data not shown). Moreover, the effects of different Mg/Ca ratios on kidney health remain unclear. Finally, we emphasized the necessity for clinical studies of the effects of mineral-balanced DSW on alleviating kidney injury caused by a high-salt diet and on kidney health.

## 4. Materials and Methods

### 4.1. Preparation of Mineral-Balanced Deep Sea Water (DSW)

DSW was obtained from the East Sea at a depth of 510 m. DSW was desalted via reverse osmosis (RO) and electrodialysis (ED) process. DSW was processed with the ‘mineral module manufacturing technology’ of Sempio Fermentation R&D Center (Cheongju, Korea), which can design target-customized mineral modules. Through this process, the DSW was manufactured into two types of samples: termed here the high magnesium low sodium (HMLS) and high magnesium high calcium (HMHC) samples. All DSW samples were provided by the Sempio Fermentation R&D Center. The mineral contents of the DSW samples are shown in Table 5.

### 4.2. Animals

The study was approved by the Institutional Animal Care and Use Committee (No. WJIACUC20191007-4-01). Male Sprague–Dawley (SD) rats were purchased from Daehan Biolink (Eumseong, Korea). After seven days of acclimatization, the experiment was conducted from eight weeks of age. Two rats were housed together in a cage. All rats were randomly divided into four groups (n = 8) consisting of control (normal diet and water), NaCl (8% NaCl diet and water), HMLS (8% NaCl diet + HMLS mineral water), HMHC (8% NaCl diet + HMHC mineral water) groups. Normal diet was sodium deficient diet TD.90228 (Harlan Inc., Indianapolis, IN, USA) and 8% NaCl diet was TD.92012 (Harlan Inc., Indianapolis, IN, USA). The rats were given free access to food and water. During the experimental period, the body weights, food intake, and water intake levels were checked five times a week. Blood pressure was measured once a week with tail cuff methods using BP-2000 series II (Visitech systems, Inc., Apex, NC, USA). The rats were placed in metabolic cages for 12 h a week and urine samples were collected. When the experiment was completed, the rats were anesthetized using an isoflurane respiratory anesthetic method.

### 4.3. Measurement of Urine Protein

Protein concentrations in the urine samples were determined according to the manual of the Rat Urinary Protein Assay Kits used here (Chondrex, Woodinville, WA, USA). Urine samples were centrifuged at 10,000 rpm for three minutes. 3% sulfosalicylic acid was added to each sample and 0.1 N HCl was added as a blank. The samples were incubated for ten minutes at room temperature and read using a plate reader at OD 450 nm.

### 4.4. Analysis of Trace Minerals in Urine

Na^+^ was measured in the urine during the fourth week using an inductively coupled plasma mass spectrometer (ICP-MS). Urine samples were analyzed after six-fold dilution.

### 4.5. Measurement of Kidney Injury Markers in Urine

Kidney injury markers were measured in the urine of the rats at four weeks. The levels of neutrophil gelatinase-associated lipocalin (NGAL), albumin, and creatinine were analyzed with an ELISA kit following the manufacturer’s protocol. NGAL ELISA assay kits (Eagle Biosciences Inc., Amherst, NH, USA), Albumin (Chondrex, Woodinville, WA, USA), and creatinine kits (Abcam, Cambridge, UK) were used.

### 4.6. Total RNA Extraction and Quantitative Reverse Transcription (qRT-PCR)

Total RNA was extracted from the kidney tissues and homogenized with 1 mL of TRIzol (Life Technologies, Rockville, MD, USA) per 100 g of tissue. cDNA was synthesized using a Transcriptor first strand cDNA synthesis kit (Life Technologies, Rockville, MD, USA) following the manufacturer’s protocol. qRT-PCR was performed. Relative mRNA expression levels were calculated using the comparative 2^−∆∆Cq^ method and were normalized with GAPDH. The primers were as follows: GPx forward 5′-tcccttgcaaccagttcg-3′ and reverse 5′-cttgaggctgttcaggatctc-3′; GSR forward 5′-ttcctcatgagaaccagatcc-3′ and reverse 5′-ctgaaagaacccatcactggt-3′; SOD1 forward 5′-ccagcggatgaagagagg-3′ and reverse 5′-ggacacattggccacacc-3′; HO-1 forward 5′-gtcaggtgtccagggaagg-3′ and reverse 5′-ctcttccagggccgtataga-3′; LRP2 forward 5′-ctcccctggagctgatga-3′ and reverse 5′-ttgggttttcgtttgaagatg-3′; Gapdh forward 5′-gcaagttcaacggcacagt-3′ and reverse 5′-gaagatggtgatgggtttcc-3′.

### 4.7. Measurement of Malondialdehyde (MDA)

MDA levels in the kidney tissues were measured according to the method of Ohkawa [51]. Kidney tissues were washed with 0.9% NaCl and homogenized in 0.9 mL of 1.15% KCl per 100 mg of tissue. 8.1% sodium dodecyl sulfate, 20% acetic acid and 0.9% thiobarbituric acid were mixed and added to a 10% tissue homogenate. Distilled water was added, and the mixture was incubated at 95 °C for one hour. After adding n-butanol: pyridine (15:1, *v*/*v*) and distilled water, the mixture was centrifuged at 4 °C at 4000× *g* for 10 min. The supernatant was moved to a 96-well plate and the absorbance levels were measured at 532 nm.

### 4.8. Measurement of Superoxide Dismutase (SOD) Activity

The SOD activity of the kidney tissue was measured using an ELISA kit (Biovision, Milpitas, CA, USA). The kidney tissues were washed with DPBS and homogenized in ice-cold 0.1 M Tris/HCl at pH 7.4 containing 0.5% Triton X-100, 5 mM β-mercaptoethanol, and 0.1 mg/mL phenylmethylsulfonyl fluoride. The tissue homogenate was centrifuged at 14,000× *g* for five minutes at 4 °C and supernatant was used for the assay. The assay was performed according to the protocol provided in the kit.

### 4.9. Statistical Analysis

All data are expressed as the mean ± standard error of the mean (SEM). Data were analyzed by means of a one-way analysis of variance (ANOVA) followed by Duncan’s multiple range test in SAS 9.4 (SAS, Cary, NC, USA). *p*-values of <0.05 were considered as statistically significant. For multiple comparisons data, Bonferroni analysis were applied (*p* < 0.05).

### 4.10. RNA-Sequencing

Total RNA was isolated using TRIzol reagent (Invitrogen, Waltham, MA, USA) in the kidneys. RNA quantification was performed using an ND-2000 spectrophotometer (Thermo Inc., Waltham, MA, USA). Libraries were prepared from the total RNA using a NEBNext Ultra II Directional RNA-Seq Kit (New England BioLabs, Inc., Ipswich, MA, USA). The mRNA isolation step was performed using a Poly(A) RNA selection kit (LEXOGEN, Inc., Wien, Austria); this was followed by cDNA synthesis and shearing following the manufacturer’s instructions. The enrichment step was carried out using PCR. Subsequently, libraries were checked using an Agilent 2100 bioanalyzer (DNA High Sensitivity Kit) to evaluate the mean fragment size. Quantification was performed using a library quantification kit with a StepOne Real-Time PCR system (Life Technologies, Inc., Carlsbad, CA, USA). High-throughput sequencing was performed as paired-end 100 sequencing using HiSeq X10 (Illumina, Inc., San Diego, CA, USA). Quality control of the raw sequencing data was carried out via FastQC. We removed adapter and low-quality reads (<Q20) using FASTX_Trimmer and BBMap. Gene expression levels were assumed using FPKM (fragments per kb per million reads) values by Cufflinks and were normalized based on the quantile normalization method using EdgeR within R. Graphic visualization was carried out using ExDEGA (E-Biogen, Inc., Seoul, Korea).

## Figures and Tables

**Table 1:** Body Weight, Food Intake, Water Intake, Kidney Weight and Blood Pressure Values of Rats.

	Group			
	CON (n = 8)	NaCl (n = 8)	HMLS (n = 8)	HMHC (n = 8)
Body Weight (g)				
Initial Weight	267.3 ± 5.9	268.4 ± 11.0	268.8 ± 10.6	267.6 ± 11.9
Final Weight	406.9 ± 15.1	393.0 ± 13.2	393.5 ± 13.2	395.5 ± 18.2
Food Intake (g/day)	25.6 ± 0.8	26.1 ± 0.6	26.1 ± 0.1	26.2 ± 0.5
Water Intake (mL/day)	49.0 ± 2.1 ^c^	113.5 ± 2.1 ^b^	149.0 ± 8.6 ^a^	139.6 ± 23.9 ^a^
Kidney Weight (2ea) (g)	2.62 ± 0.26 ^b^	3.07 ± 0.31 ^a^	3.09 ± 0.16 ^a^	3.03 ± 0.16 ^a^
Blood Pressure (mmHg)	113.1 ± 4.6 ^b^	161.8 ± 6.0 ^a^	161.1 ± 6.3 ^a^	160.7 ± 3.7 ^a^

CON, normal diet + distilled water; NaCl, 8% NaCl diet + distilled water; HMLS, 8% NaCl diet + high magnesium low sodium water; HMHC, 8% NaCl diet + high magnesium high calcium water. Different letters above the data indicate significant differences (Duncan’s multiple range test; *p* < 0.05).

**Table 2:** Renal-System-Related Gene Expressions in the NaCl vs. HMLS Group.

Gene ID	Gene Name	Fold Change
*Down-regulated*		
Hbb	Hemoglobin Subunit beta	0.238
Nphs2	nephrosis 2, idiopathic, steroid-resistant	0.240
Tbx18	T-box18	0.282
Col4a3	collagen type IV alpha 3 chain, transcript variant X1	0.292
Myocd	myocardin, transcript variant X2	0.340
Apc	APC, WNT signaling pathway regulator, transcript variant X1	0.377
Col4a4	collagen type IV alpha 4 chain	0.386
Adamts1	ADAM metallopeptidase with thrombospondin type 1 motif, 1	0.392
Tsc1	tuberous sclerosis 1, transcript variant X1	0.401
Fat4	FAT atypical cadherin 4, transcript variant X1	0.457
Ctnnd1	catenin delta 1, transcript variant X6	0.477
Ahi1	Abelson helper integration site 1	0.480
Lrrk2	leucine-rich repeat kinase 2, transcript variant X1	0.484
Hoxc11	homeobox C11	0.487
Vegfa	vascular endothelial growth factor A, transcript variant 1	0.498

**Table 3:** Response to Oxidative-Stress-Related Gene Expression in the NaCl vs. HMLS Group.

Gene ID	Gene Name	Fold Change
*Down-regulated*		
Hmox1	Heme Oxygenase 1	0.062
Hspb1	Heat Shock Protein Family B (Small) Member 1	0.092
Nr4a3	Nuclear Receptor Subfamily 4 Group A Member 3	0.099
Jun	Jun Proto-Oncogene, AP-1 Transcription Factor Subunit	0.142
Hbb	Hemoglobin Subunit beta	0.238
Klf6	Kruppel Like Factor 6	0.323
Arntl	Aryl Hydrocarbon Receptor Nuclear Translocator-like, transcript variant X4	0.375
Uaca	Uveal Autoantigen with Coiled-coil domains and Ankyrin repeats, transcript variant X1	0.400
ll1r1	Interleukin 1 Receptor type 1, transcript variant X8	0.407
Dusp1	Dual Specificity Phosphatase 1	0.438
Egfr	Epidermal Growth Factor Receptor	0.440
Abcc2	ATP binding cassette subfamily C member 2	0.467
Lrrk2	Leucine-rich repeat kinase 2, transcript variant X1	0.484

**Table 4:** DAVID Functional Analysis and KEGG pathway Results of DEGs in the HMLS vs. NaCl Group.

Category	Term	ID	*p* Value	Benjamini
GO, cellular component	Hemoglobin complex	GO:0005833	4.4 × 10^−5^	3.4 × 10^−3^
	Perinuclear region of cytoplasm	GO:0048471	4.5 × 10^−5^	3.4 × 10^−3^
	Extracellular exosome	GO:0070062	3.6 × 10^−4^	1.8 × 10^−2^
GO, biological process	Negative regulation of inclusion body assembly	GO:0090084	1.1 × 10^−5^	8.0 × 10^−3^
	Oxygen transport	GO:0015671	7.4 × 10^−5^	2.2 × 10^−2^
	Negative regulation of neuron apoptotic process	GO:0043524	9.0 × 10^−5^	2.2 × 10^−2^
GO, molecular function	Unfolded protein binding	GO:0051082	2.0 × 10^−6^	4.8 × 10^−4^
	Chaperone binding	GO:0051087	3.8 × 10^−5^	4.7 × 10^−3^
	Oxygen transporter activity	GO:0005344	8.9 × 10^−5^	7.2 × 10^−3^
KEGG pathways	MAPK signaling pathway	−	4.1 × 10^−4^	4.6 × 10^−2^
	African trypanosomiasis	−	1.1 × 10^−3^	5.7 × 10^−2^
	Estrogen signaling pathway	−	1.7 × 10^−3^	5.7 × 10^−2^

**Table 5:** Mineral contents of the water sample.

Sample	Mineral Contents (ppm)				Na/Mg	Mg/Ca
	Na^+^	Mg^2+^	K^+^	Ca^2+^		
DSW	10700	1380	480	390	7.75	3.5
HMLS	36	179	1.7	41.5	0.2	4.3
HMHC	133	211	48	200	0.63	1.1

DSW, deep sea water; HMLS, high magnesium low sodium; HMHC, high magnesium high calcium. Na^+^, Mg^2+^, K^+^ and Ca^2+^ ions were measured in the DSW and water samples. The ratio of Na/Mg and Mg/Ca is shown in the table.

## Data Availability

Not applicable.
